# Supplementary material for: K-Ras controls asymmetric cell divisions from the primary cilium
Source: Cell Death Dis. 2026 Jul 11;17(1):819. doi: 10.1038/s41419-026-09089-y (PMC13594257; doi:10.1038/s41419-026-09089-y)
Supplement: Supplementary file 1 [file 41419_2026_9089_MOESM1_ESM.pdf]

Supplementary Information

Supplementary Figures

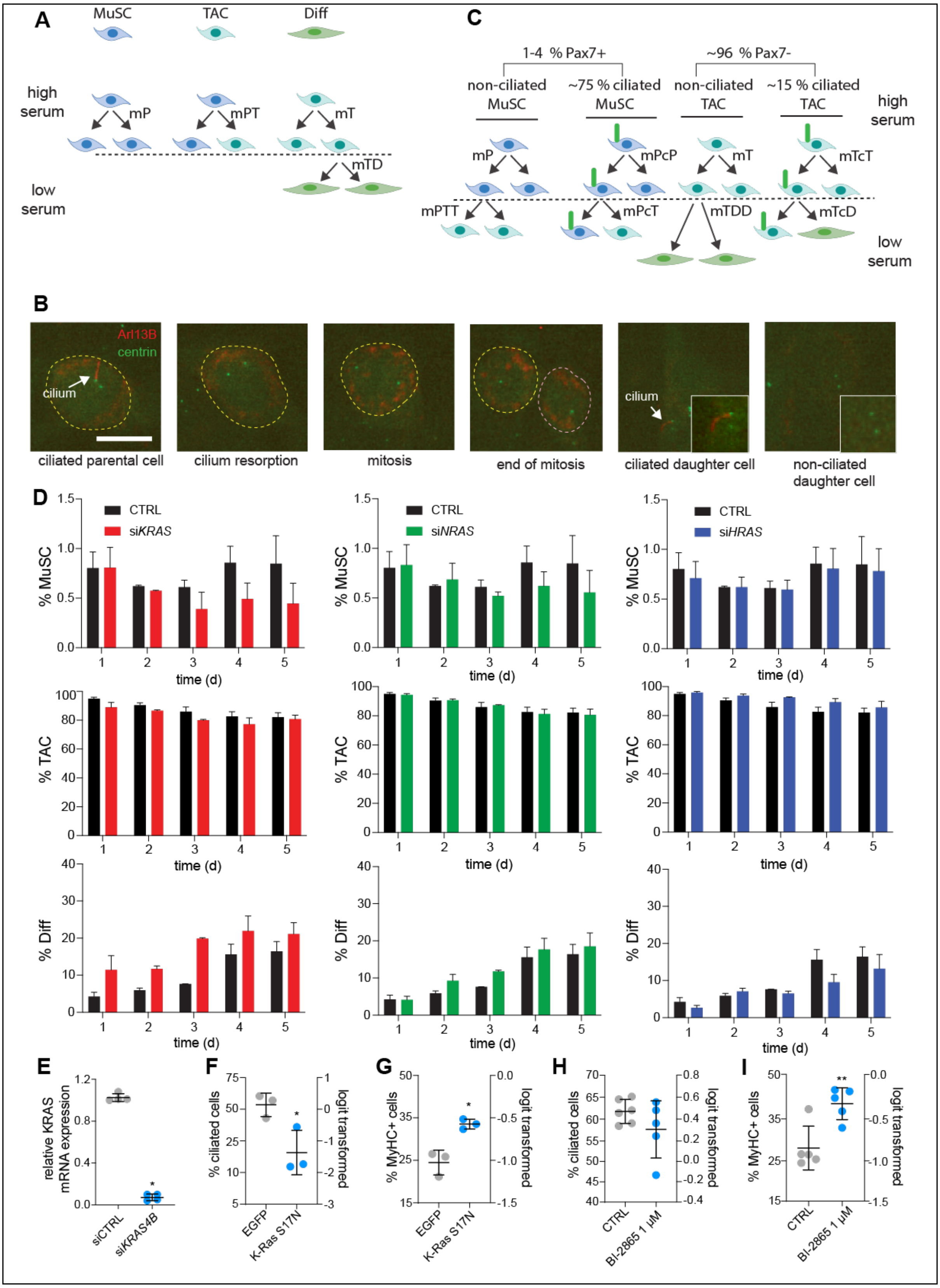

Figure S1. Related to Figure 1.

(**A**) Schematic of mouse skeletal muscle C2C12 cell hierarchy with rate constants used for the first ordinary differential equation model (model 1). The decay/ death rate d is not displayed, as it impacts equally on all states. This simple model could adequately describe the evolution of MuSC and TAC in high serum, where cells proliferate. It likewise provided a good fit to data from low serum conditions, where a very low percentage of MuSC and a high percentage of TAC decrease, while differentiated cells (Diff) increase.

(**B**) Confocal images of time-lapse recording of a C2C12 cell grown in high serum for 48 h after transfection with Arl13B-mCherry as ciliary marker (arrow) and EGFP-centrin1 to mark the centrioles. Scale bar = 10 μm. The corresponding video is provided as **Video S1**.

(**C**) Schematic of C2C12 cell hierarchy with rate constants used for the second ordinary differential equation model (model 2), which included ciliation as determinant for asymmetric cell divisions. The decay/ death rate d is not displayed, as it impacts equally on all states.

(**D**) Flow-cytometric quantification of C2C12 cell sub-populations, MuSC (Pax7+/MyHC-), TAC (Pax7-/MyHC-) and Diff cells (Pax7-/MyHC+) after induction of differentiation in low serum. Indicated knockdowns of Ras isoforms were done on day 0 using 100 nM siRNAs, N = 3. Means ± SD are plotted. The time-aggregated data were published by us before [1].

(**E**) RT-PCR based *KRAS* mRNA levels normalized to *GAPDH* mRNA of C2C12 cells grown in low serum for 72 h after transfection with siCTRL or si*KRAS4B* (each 100 nM), N = 4. Means ± SD are plotted. Statistical analysis was done with the Mann-Whitney test.

(**F**) Confocal imaging-based quantification of ciliation of C2C12 cells grown in high serum for 48 h after transfection with EGFP alone or EGFP-K-RasS17N, N = 3, n = 250. Means ± SD are plotted. Statistical analysis was done with the unpaired t-test with Welch's correction.

(**G**) Flow cytometric quantification of MyHC terminal differentiation marker expression of C2C12 grown in low serum for 72 h and transfected as in (F), N = 3. Means ± SD are plotted. Statistical analysis was done with the unpaired t-test with Welch's correction.

(**H**) Confocal imaging-based quantification of ciliation of C2C12 grown in low serum for 72 h and treated with CTRL (DMSO 0.1 % in low serum medium) or pan-K-Ras inhibitor BI-2865 at the indicated concentration, N = 3, n = 250. Means ± SD are plotted. Statistical analysis was done with the unpaired t-test with Welch's correction.

(**I**) Flow cytometric quantification of MyHC terminal differentiation marker expression in C2C12 treated as in (H), N = 3. Means ± SD are plotted. Statistical analysis was done with the unpaired t-test with Welch's correction.

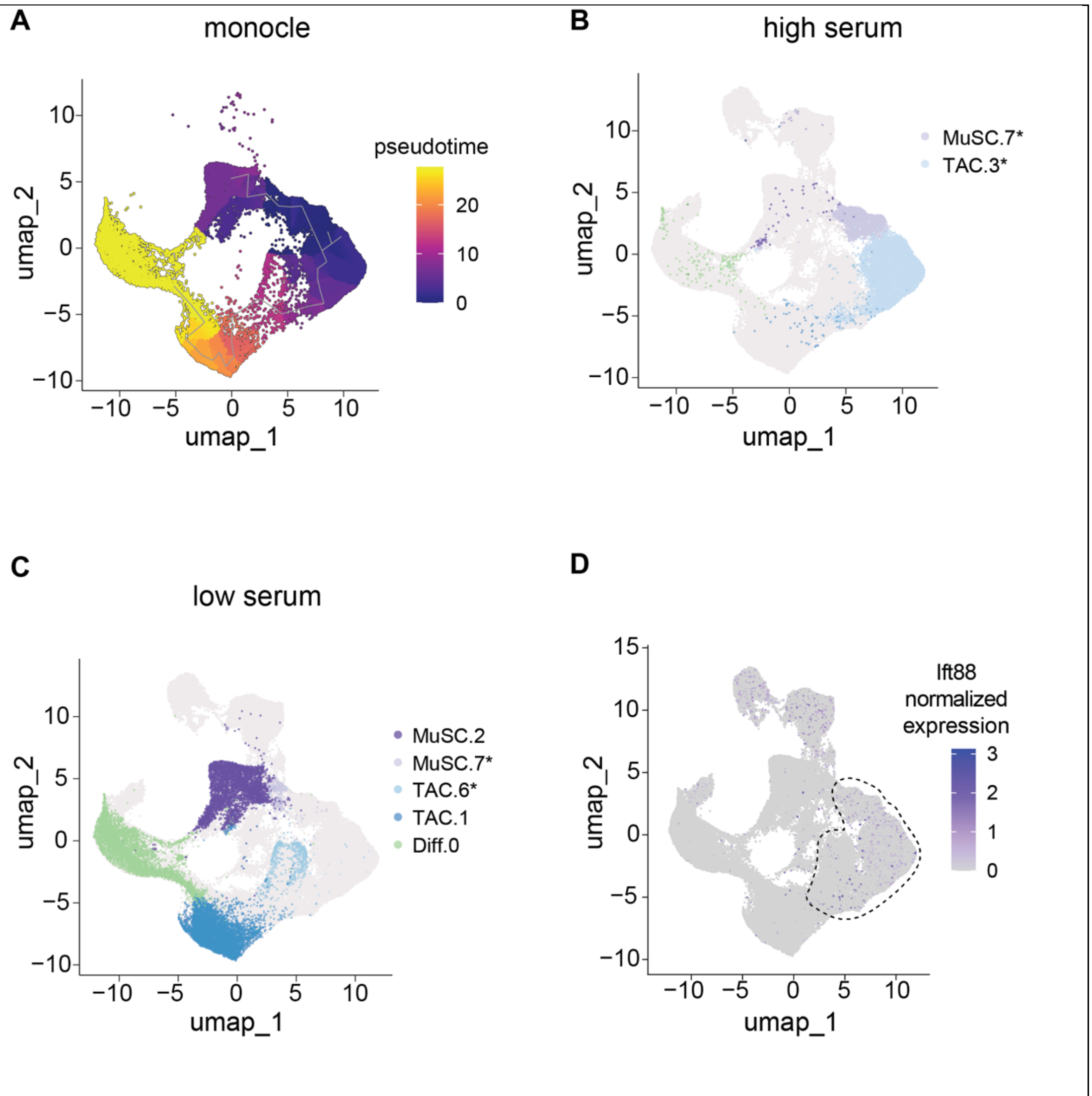

Figure S2. Related to Figure 2.

(**A**) Prediction of mouse skeletal muscle C2C12 cell differentiation trajectory and pseudotime with Monocle.

(**B, C**) UMAP projection of C2C12 cell scRNAseq data, with cells from high serum (B) and from samples of cells cultured 72 h in low serum (C) colored by cluster. Bona fide ciliated cell clusters MuSC.7, TAC.3 and TAC.6 are marked with an asterisk.

(**D**) Ift88-expression overlaid on UMAP projection of C2C12 cell scRNAseq data. Ciliated cell clusters MuSC.7, TAC.3 and TAC.6 are outlined with a dashed line.

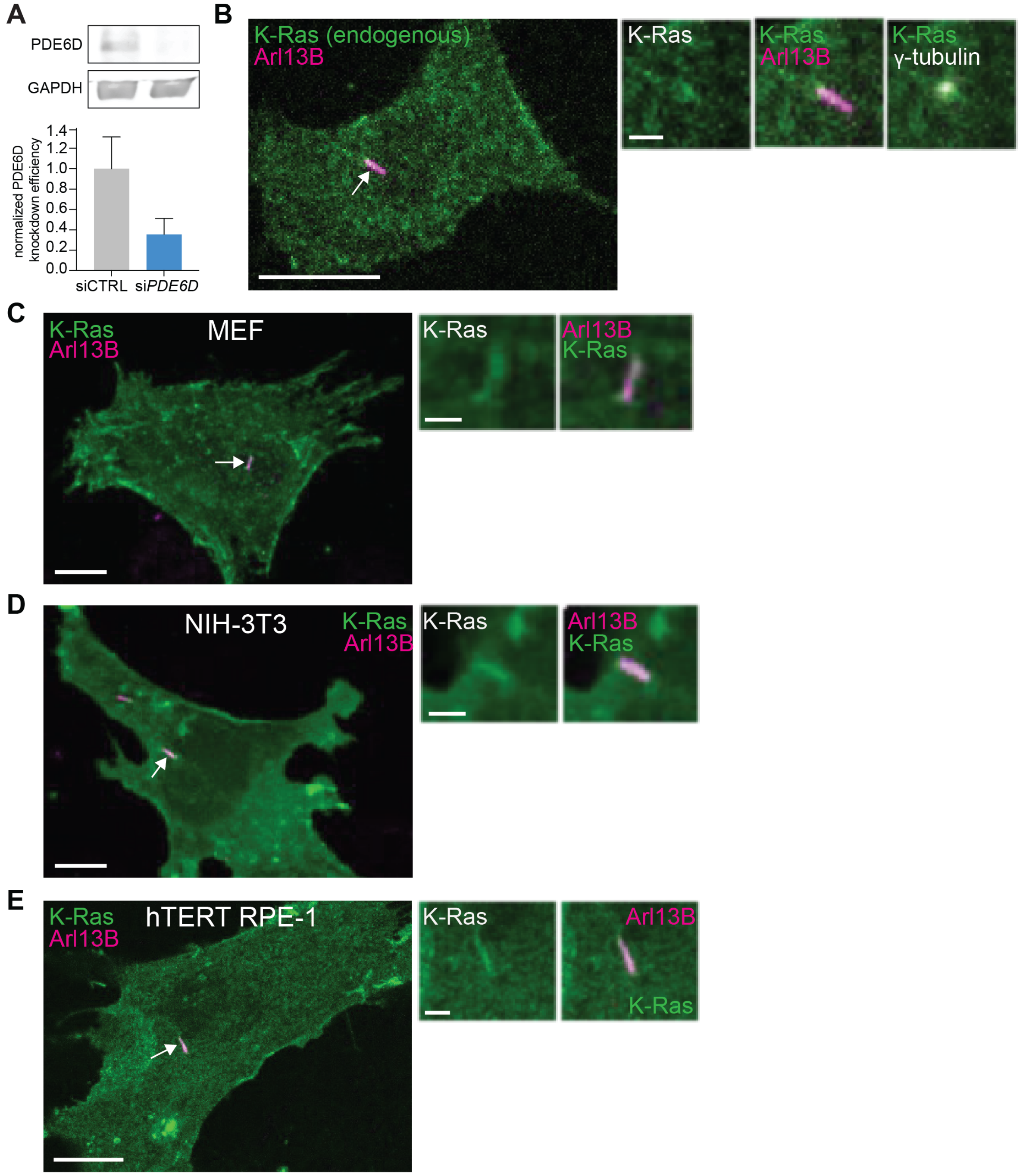

Figure S3. Related to Figure 3.

(**A**) Representative immunoblots and quantification of protein levels normalized to GAPDH of C2C12 cells grown in low serum for 72 h after transfection with 100 nM siCTRL or 100 nM si*PDE6D*, N = 3. Means ± SD are plotted. Statistical analysis was done with the Mann-Whitney test.

(**B**) Confocal image of mEos3.2-KRAS knock-in C2C12 cells cultured in high serum for 24 h directly after FACS-sorting and immunolabelled for ciliary marker Arl13B (arrow) and centriolar marker γ-tubulin. Scale bar = 10 µm. Smaller images to the right show enlargement of the area containing the cilium. Scale bar = 2 µm.

(**C-E**) Confocal images of MEF wt (B), NIH-3T3 (C) and hTERT RPE-1 cells (D) that were transfected with mEGFP-K-Ras. 24 h after transfection, cells were serum starved for 24 h to induce the formation of a primary cilium and immunolabelled for ciliary marker Arl13B (arrow). Scale bar = 10 µm. Smaller images to the right show enlargement of the area containing the cilium. Scale bar = 2 µm.

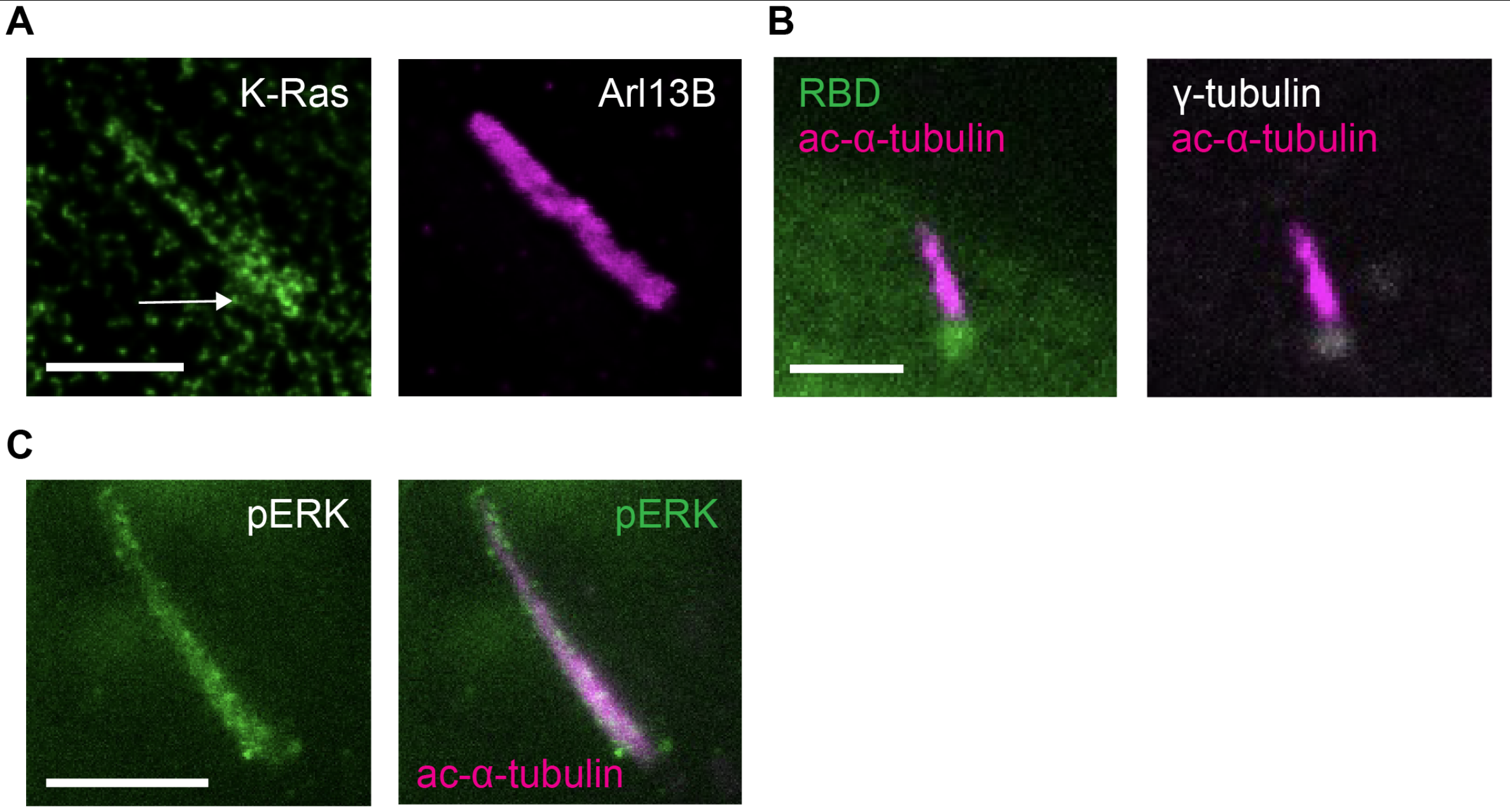

Figure S4. Related to Figure 4.

(**A**) STED images of C2C12 cells grown in high serum for 48 h after transfection with GFP2-K-Ras and immunolabeled for GFP with ATTO647N-conjugated nanobody and ciliary membrane marker Arl13B. At the base of the cilium, K-Ras appears to be distributed in a radially symmetrical fashion (arrow), as would be expected from its association with the centrioles. Scale bar = 2 μm.

(**B**) Confocal images of C2C12 cells grown in high serum for 48 h after transfection with mCherry-C-Raf-RBD and immunolabelled for acetylated α-tubulin marking the cilium. Cells were not transfected with K-Ras variant constructs, thus showing dependence on endogenous active Ras. Scale bar = 2 μm.

(**C**) Confocal images of C2C12 cells grown in high serum for 48 h. Cells were not co-transfected with K-Ras variant constructs. Immunolabelling for endogenous phospho-Thr202/Tyr204-ERK1/2 and acetylated α-tubulin. Scale bar = 2 μm.

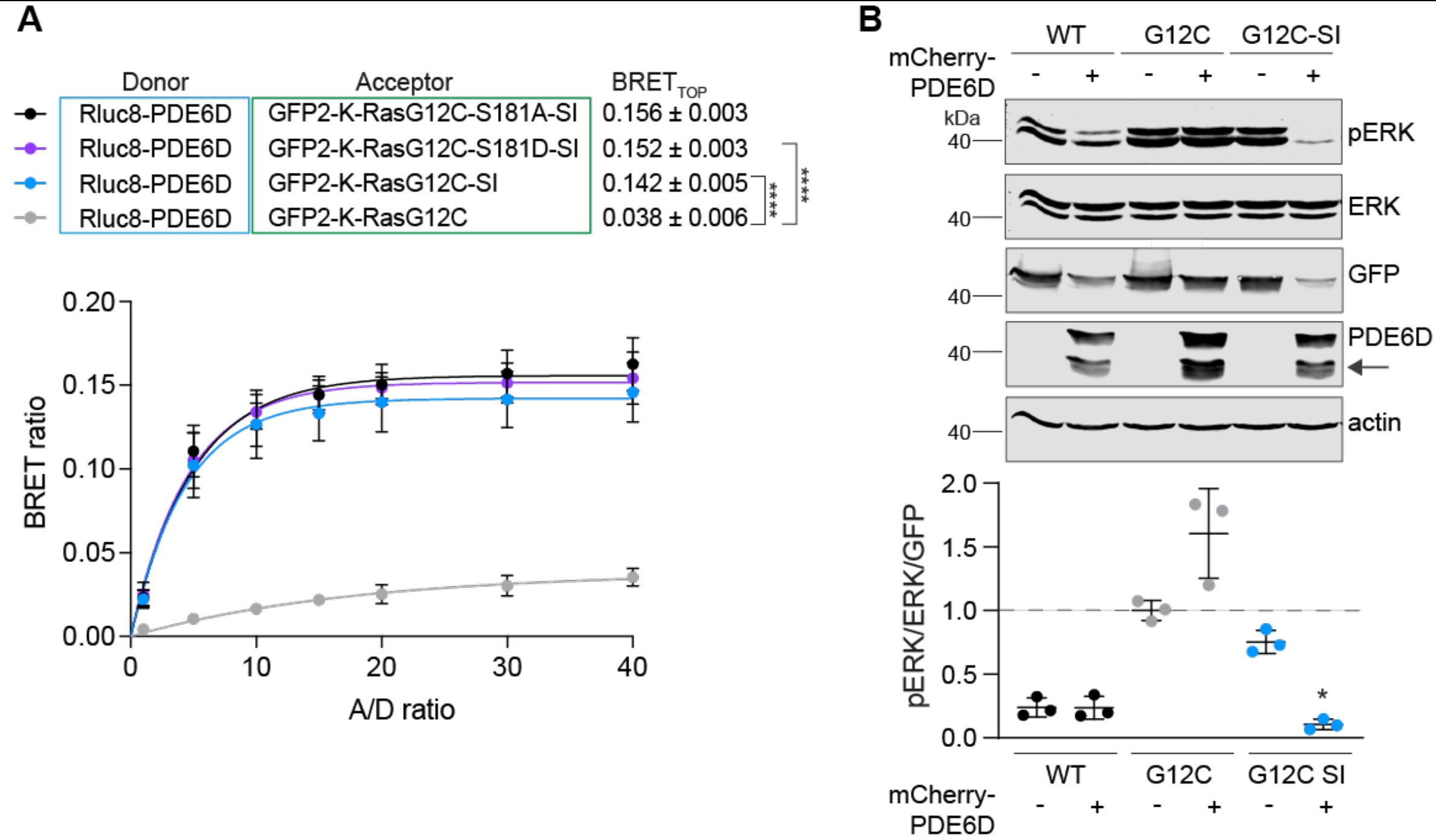

Figure S5. Related to Figure 5.

(**A**) BRET titration curves of Rluc8-PDE6D and GFP2-K-RasG12C mutants expressed in HEK cells, N = 3. Statistical analysis was performed with the extra sum-of-squares F-test.

(**B**) Cropped representative immunoblot images with quantified normalized phosphorylated ERK (pERK) levels after expression of EGFP-K-Ras (WT), EGFP-K-RasG12C (G12C) or EGFP-K-RasG12C-SI (G12C-SI) expressed in HEK cells with or without co-expression of mCherry-PDE6D at 1:1 DNA ratio for 24 h, N = 3. Means ± SD are plotted. Employed antibody labelings are indicated to the right of the blots. Statistical analysis was done using the Kruskal-Wallis test with multiple comparisons with G12C without mCherry-PDE6D expression used as a reference. Arrow marks truncated/ cleaved protein consistently observed following transfection of mCherry-PDE6D.

Video S1

C2C12 cells were transfected with Arl13B-mCherry and mEGFP-centrin. A cilium as well as the mother and daughter centrioles are clearly observed at t = 00:00:00 in the cell marked with a circle. The cilium is resorbed at t = 01:00:00 and the cell enters mitosis at approximately t = 02:00:00. The two daughter cells subsequently generated are marked with yellow and pink circles at t = 02:50:00. Both daughter cells then traverse for a short distance and only one, indicated with a pink border assembles a cilium at t = 08:50:00. This indicates asymmetric inheritance of the cilium in C2C12 cell divisions. Images were acquired at a time interval of 10 min and 29 z-stacks were acquired with a spacing of 0.5 µm. Time-lapse movie represents z-projection at maximum intensity.

Table S1: Materials and equipment employed in the study

REAGENT or RESOURCE	SOURCE	IDENTIFIER
Antibodies		
Mouse monoclonal anti-Pax7 (Pax7); Dilution 1:500 immunofluorescence (IF)	Bio-Techne	Cat# MAB1675 RRID: AB_2159833
Rabbit polyclonal anti-Arl13B; Dilution 1:500 IF	Proteintech	Cat# 17711-1-AP RRID: AB_2060867)
Alexa Fluor 647 Phalloidin; Dilution 1:50 IF	Invitrogen	Cat# A-22287 RRID: AB_2620155
ChromoTek GFP-Booster ATTO647N; Dilution 1:200 IF	Proteintech	Cat# gba647n RRID: AB_2629215
Mouse monoclonal anti-alpha tubulin (acetyl K40) [EPR16772]; Dilution 1:500 IF	Abcam	Cat# ab179484 RRID: AB_2890906
Rabbit recombinant monoclonal anti-gamma-2- tubulin antibody [EPR16793]; Dilution 1:500 IF	Abcam	Cat# ab179503 RRID: AB_2904198
Rabbit polyclonal anti-Phospho MEK1/2 (Ser217/221); Dilution 1:200 IF	Cell Signaling	Cat# 9121 RRID: AB_331648
Rabbit polyclonal anti-Phospho-p44/42 MAPK (Erk1/2) (Thr202/Tyr204) Antibody Dilution 1:200 IF	Cell Signaling	Cat# 9101 RRID: AB_331646
Goat anti-Rabbit IgG (H+L) cross-adsorbed secondary antibody, Alexa Fluor 594; Dilution 1:200 IF	ThermoFisher Scientific	Cat# A-11012 RRID: AB_2534079
Goat anti-mouse IgG (H+L) cross-adsorbed secondary antibody, Alexa Fluor 647; Dilution 1:200 IF	ThermoFisher Scientific	Cat# -21235 RRID: AB_2535804

Anti-Rabbit IgG - Atto 633 antibody produced in goat<br>Dilution 1:200 IF	Merck	Cat# 41176<br><br>RRID: AB_1137641
Mouse monoclonal anti-myosin 4 antibody (MF20), eFluor660, eBioscience;<br>Dilution 1:100 Flow cytometry	ThermoFisher Scientific	Cat# -50-6503-80<br>RRID: AB_2574266
Rabbit polyclonal anti-p44/42 MAPK (Erk1/2);<br>Dilution 1:1 000 Western Blot (WB)	Cell Signaling	Cat# 9102<br>RRID: AB_330744
Mouse monoclonal anti-phospho-p44/42 MAPK (Erk1/2) (Thr202/Tyr 204) (E10);<br>Dilution 1:2 000 WB	Cell Signaling	Cat# 9106<br>RRID: AB_331768
Mouse monoclonal anti-PDE6D (E7);<br>Dilution 1:1 000 WB	Santa Cruz Biotechnology	Cat# sc-166854<br>RRID: AB_2161460
Rabbit polyclonal anti-GFP antibody;<br>Dilution 1:5 000 WB	Merck	Cat# SAB4301138<br>RRID: AB_2750576
Mouse monoclonal anti-beta actin antibody;<br>Dilution 1:5 000 WB	Merck	Cat# A5441<br>RRID: AB_476744
IRDye 680RD goat anti-Rabbit IgG;<br>Dilution 1:10 000 WB	LI-COR Biosciences	Cat# 926-68071<br>RRID: AB_10956166
IRDye 680LT donkey anti-Mouse IgG;<br>Dilution 1:10 000 WB	LI-COR Biosciences	Cat# 926-68022<br>RRID: AB_10715072
IRDye 800CW goat anti-Rabbit IgG;<br>Dilution 1:10 000 WB	LI-COR Biosciences	Cat# 926-32211<br>RRID: AB_621843
IRDye 800CW donkey anti-Mouse IgG;<br>Dilution 1:10 000 WB	LI-COR Biosciences	Cat# 926-32212<br>RRID: AB_621847
Bacterial and virus strains		
E. coli DH10B	New England BioLabs	Cat# C3019I
Biological samples		
N/A	N/A	N/A

Chemicals, peptides, and recombinant proteins		
BI-2865	Selleckchem	Cat# E1474
Dimethyl sulfoxide (DMSO)	PanReac AppliChem	Cat# A3672
Hoechst 33342	ThermoFisher Scientific	Cat# 62249
Critical commercial reagents		
Gateway LR Clonase II enzyme mix	ThermoFisher Scientific	Cat# 11791020
Gateway BP Clonase II enzyme mix	ThermoFisher Scientific	Cat# 11789020
Bio-Rad Protein Assay Kit II	Bio-Rad	Cat# 5000002
Pierce Protease Inhibitor Tablets	ThermoFisher Scientific	Cat# A32963
PhosSTOP	Merck	Cat# 4906845001
Lipofectamine RNAiMAX Transfection Reagent	ThermoFisher Scientific	Cat# 13778075
Lipofectamine 2000 Transfection Reagent	ThermoFisher Scientific	Cat# 11668019
jetPRIME transfection reagent	Polyplus	Cat#101000046
Coelenterazine 400a; 2,8-Dibenzyl-6-phenylimidazo[1,2a]pyrazin-3-(7H)-one; DeepBlueC	Gold Biotechnology	Cat# C-320-1
NucleoSpin RNA Plus, Mini kit for RNA purification	Macherey-Nagel	Cat# 740984.250
SuperScriptIII Reverse Transcriptase	ThermoFisher Scientific	Cat# 18080093
SsoAdvanced Universal SYBR Green Supermix	Bio-Rad	Cat# 1725274
Rapid DNA ligation kit	ThermoFisher Scientific	Cat# K1422
Shrimp Alkaline Phosphatase (rSAP)	New England Biolabs	Cat# M0371S
BsrGI-HF	New England Biolabs	Cat# R3575S
NheI-HF	New England Biolabs	Cat# R3131S
NotI	New England Biolabs	Cat# R0189S
SpeI-HF	New England Biolabs	Cat# R3133S
PstI	New England Biolabs	Cat# R0140S
XbaI	New England Biolabs	Cat# R0145S
SalI	New England Biolabs	Cat# R0138S
NEBuffer r2.1	New England Biolabs	Cat# B6002S
NEBuffer r3.1	New England Biolabs	Cat# B6003S
rCutSmart Buffer	New England Biolabs	Cat# B6004S

Q5 High fidelity DNA polymerase	New England Biolabs	Cat# M0491L
dNTP set, 100 mM	ThermoFisher Scientific	Cat# R0181
mMessage mMachine SP6 transcription kit	ThermoFisher Scientific	Cat# AM1340
Agarose	Merck	Cat# 2120
NucleoSpin Gel and PCR clean-up kit, mini kit for gel extraction or PCR clean-up	Macherey-Nagel	Cat# 740609.50
Experimental models: Cell lines		
Mouse cell line, C2C12	ATCC	Cat# CRL-1772 RRID: CVCL_0188
Mouse cell line, C2C12-mEos3.2-KRAS knock-in	Synthego	N/A
Human cell line, HEK293 c18	ATCC	Cat# CRL-10852, RRID: CVCL_6974
Mouse cell line, wt MEFs	ATCC	Cat# CRL-2991, RRID: CVCL_L69 0
Mouse cell line, NIH-3T3	ATCC	Cat# CRL-1658 RRID: CVCL_0594
Human cell line, hTERT RPE-1	ATCC	Cat# CRL-4000 RRID:CVCL_4388
Experimental models: Organisms/strains		
N/A	N/A	N/A
Oligonucleotides		
Custom qPCR primer: KRAS mouse_F 5’-3’sequence GGAGTACAGTGCAATGAGGGAC	Genecust	N/A
Custom qPCR primer: KRAS mouse_R 5’-3’sequence CCAGGACCATAGGCACATCTTC	Genecust	N/A
Custom qPCR primer: GAPDH mouse_F 5’-3’ sequence TGGTGAAGGTCGGTGTGAA	Eurogentec	N/A
Custom qPCR primer: GAPDH mouse_R 5’-3’ sequence ATGAAGGGGTCGTTGATGG	Eurogentec	N/A

Custom Restriction cloning primer: PstI KRAS S17N Forward<br>5'-3' sequence: TCACTGCAGGCATGACTGAATATAAACTTGTGGTAGTTG	Integrated DNA technologies	N/A
Custom Restriction cloning primer: SpeI KRAS S17N Reverse<br>5'-3' sequence: CTGACTAGTTTACATAATTACACACTTTGTCTTTGACT	Integrated DNA technologies	N/A
Custom Restriction cloning primer: NotI KRAS SI Forward<br>5'-3' sequence: GTGGCGGCCGCATGGTGAGCAAGGGCG	Integrated DNA technologies	N/A
Custom Restriction cloning primer: SpeI KRAS SI Reverse<br>5'-3' sequence: ACTACTAGTTCAAGAAACGGAGCAGATGG	Integrated DNA technologies	N/A
Human Negative control siRNA	QIAGEN	Cat# 1027310
Human *HRAS* siRNA (FlexiTube siRNA)<br>5'-3'sequence<br>CGGAAGCAGGUGGUCAUUA	QIAGEN	Cat# 1027417
Mouse *KRAS* siRNA (targeting both *KRAS*4A and 4B transcripts; si-pan *KRAS*)<br>(ON-TARGETplus SMARTpool siRNA)<br>5'-3' sequences<br>GAACAGUAGACACGAAAA<br>AGCAAGGAGUUACGGAUU<br>GGUUGGAGCUGGUGGCGUA<br>GGUGUACAGUUAUGUGAAU	Horizon Discovery	Cat# L-043846-01-0005
Custom siRNA: Mouse *NRAS* siRNA	Horizon Discovery	N/A

5’-3’ sequence GCAAAUUAAGCGUGUGAAAUUU		
Custom siRNA: Mouse *KRAS4B* siRNA 5’-3’ sequence Cy3-AAGAAGAAGUCAAGGACAAUU	Horizon Discovery	N/A
Mouse *PDE6D* siRNA (ON-TARGETplus SMARTpool siRNA) 5’-3’ sequences UCUCAUACCUUUAACUUGU CGCCUGAGUCCCAGAUGAU CAAAGGACAAUGCCUAGAA GCGCAAACAUGGGAACAAA	Horizon Discovery	Cat# L-062279-01-0005
Recombinant DNA		
C453-E04: attB4-CMV51p>-attB5 (L4 L5)	FNL Combinatorial Cloning Platform, RAS-Initiative	Addgene# 162973
C413-E36: attB4-CMV51p>-attB1r (L4 R1)	FNL Combinatorial Cloning Platform, RAS-Initiative	Addgene# 162927
pDest-305 (attR4-attR2)	FNL Combinatorial Cloning Platform, RAS-Initiative	Addgene# 161895
pDest-312 (attR4-attR3)	FNL Combinatorial Cloning Platform, RAS-Initiative	Addgene# 161897
C512-E01: attB5r-meGFP-nostop-attB1r (R5 R1)	FNL Combinatorial Cloning Platform, RAS-Initiative	Addgene# 162940
C511-E03: attB5r-Rluc8-nostop-attB1r (R5 R1)	[2]	N/A
pDONR235 GFP2-no stop R5 R1	Generated by Genewiz Inc.	N/A
C512-E04: attB5r-mCherry-nostop-attB1r (R5 R1)	FNL Combinatorial Cloning Platform, RAS-Initiative	Addgene# 162941

C232-E06: attB2r-mCherry-attB3 (R2 L3)	FNL Combinatorial Cloning Platform, RAS-Initiative	Addgene# 162903
Gateway pDONR221 vector	Thermofisher Scientific	Cat# 12536017
Hs. PDE6D	[2]	N/A
Hs. KRAS4B	RAS mutant collection V2.0, RAS-Initiative	Addgene# 83129
Hs. HRAS WT	RAS mutant collection V2.0, RAS-Initiative	Addgene# 83181
Hs. KRAS4B-S181A	RAS mutant collection V2.0, RAS-Initiative	Addgene# 83136
Hs. KRAS4B-S181D	RAS mutant collection V2.0, RAS-Initiative	Addgene# 83137
Hs. BRAF	RAS pathway clone collection V2.0, RAS-Initiative	Addgene# 70299
pDest305-CMV-GFP2-K-Ras4B	This paper	N/A
pDest305-CMV- GFP2-H-Ras	This paper	N/A
pDest305-CMV- GFP2-N-Ras	This paper	N/A
pDest305-CMV- Rluc8-PDE6D	This paper	N/A
pDest305-CMV- mCherry-PDE6D	This paper	N/A
pDest305-CMV- GFP2-B-Raf	This paper	N/A
pDest305-CMV- mCherry-B-Raf	This paper	N/A
pmEGFP-N-Ras	[3]	N/A
pDest305-CMV-mEGFP-K-Ras4B	[1]	
pDest305-CMV-mEGFP-K-Ras4B G12C	[1]	
K-Ras-SI g-block	Integrated DNA technologies	N/A
pDest305-CMV-GFP2-K-Ras4B-SI	This paper	N/A
pmEGFP-K-Ras SI	Generated by Genecust	N/A
pDest305-CMV-GFP2-K-Ras4B G12C-SI	Generated by Genecust	N/A
pDest305-CMV-GFP2-K-Ras4B G12C S181A-SI	Generated by Genecust	N/A
pDest305-CMV-GFP2-K-Ras4B G12C S181D-SI	Generated by Genecust	N/A
pEGFP-C1	NovoPro	Cat# V012024

pcDNA3.1(+)mCherry-K-Ras4B S17N	[4]	N/A
pEGFP-K-Ras4B S17N	This paper	N/A
pCS2+-CMV-mEGFP-K-Ras4B-wt	This paper	N/A
pCS2+-CMV-mEGFP-K-Ras4B-SI	This paper	N/A
pCS2+-CMV-mEGFP-K-Ras4B-S17N	This paper	N/A
pcDNA3.1(+)	Invitrogen	Cat# V79020
pmRFP1-C-RBD	[3]	N/A
pEGFP-C1-Centrin	Addgene	Cat# 72641
Mouse Arl13B codon optimized g-block	Integrated DNA technologies	N/A
pDest305-CMV-Arl13B-mCherry	This paper	N/A
Software and algorithms		
GuavaSoft version4.0	Cytek Biosciences	N/A
Leica Application Suite X version 4.12	Leica Microsystems	RRID: SCR_013673
GraphPad Prism version 9.5.1	GraphPad	RRID: SCR_002798
Fiji version 2.9.0	[5]	RRID: SCR_002285
FlowFate version 1.2	[6]	N/A
Image Studio software version 5.2	LI-COR Biosciences	N/A
Bio-Rad CFX Manager version 3.1	Bio-Rad	N/A
FlowJo version 10.8.2	FlowJo LLC	RRID:SCR_008520
OriginPro version 2021	OriginLab	RRID:SCR_014212
SnapGene version 8.0.2	SnapGene	RRID:SCR_015052
Cell Ranger version 8.0.1	10x Genomics	RRID:SCR_017344
R version 4.3	R Core Team	RRID: SCR_003005
Seurat version 5.0.1	[7]	RRID:SCR_016341
Monocle3 version 1.3.7	[8]	RRID:SCR_018685

MAST	[9]	RRID:SCR_016340
Clustree	[10]	RRID:SCR_016293
Louvain algorithm	[11]	N/A
Python version 3.13.3	Python software foundation	https://www.python.org/ RRID:SCR_008394
Fitting-curves-python: curve fitting algorithm	This paper	https://github.com/GesaHlzer/BachelorThesis
**Other**		
Guava easyCyte 6HT 2L flow cytometer	Cytek Biosciences	Cat# 0500-4007
Odyssey CLx Infrared Imaging System	LI-COR Biosciences	Cat# LI9140-00
CFX-connect real-time PCR Detection System	Bio-Rad	Cat# 1855200
4D-Nucleofector® X Unit	Lonza	Cat# AAF-1003X
Qubit 4 Fluorometer	ThermoFisher Scientific	Cat# Q33238
NextSeq2000 sequencing system	Illumina	Cat# 20038897
5200 Fragment Analyzer 12 capillary electrophoresis system	Agilent	Cat# M5310AA
SE Cell Line 4D-Nucleofector™ X Kit L	Lonza	Cat# V4XC-1024
Chromium fixed RNA profiling reagent kit, mouse transcriptome	10x Genomics	Cat# PN-1000496
dsDNA Quantitation kit, HS	ThermoFisher Scientific	Cat# Q32854
NextSeq™ 2000 P4 XLEAP-SBS™ Reagent Kit	Illumina	Cat# 20100994
Dulbecco's Modified Eagle's Medium (DMEM), high glucose	ThermoFisher Scientific	Cat# 11965092
Dulbecco's Modified Eagle Medium/Nutrient Mixture F-12 (DMEM/F-12)	ThermoFisher Scientific	Cat# 11320033
DMEM, high glucose, no glutamine, no phenol red	ThermoFisher Scientific	Cat# 31053028
Fetal bovine serum (FBS)	ThermoFisher Scientific	Cat# 10270106

Horse serum (HS)	ThermoFisher Scientific	Cat# 16050130
L-glutamine (200 mM)	ThermoFisher Scientific	Cat# A2916801
Penicillin-Streptomycin (10,000 U/mL)	ThermoFisher Scientific	Cat# 10378016
Hygromycin B (50mg/mL in PBS)	Takara Bio	Cat# 631309
Opti-MEM Reduced Serum Medium	ThermoFisher Scientific	Cat# 31985047
Paraformaldehyde 16 % (w/v)	Avantor	Cat# 30525-89-4
Bovine Serum Albumin	AppliChem	Cat# A6588
TWEEN 20	Merck	Cat# P9416
Triton X-100	Merck	Cat# T8787
Semi-dry transfer kit	Bio-Rad	Cat # 1704272
VECTASHIELD Anti-fade mounting medium	Vector Laboratories	Cat# H-1000-10
MOWIOL 4-88 reagent	Merck	Cat# 475904
Cellview cell culture dish, polystyrene, 35/10 mm, glass bottom	Greiner	Cat# 627860
VWR 6-well cell culture plate	Avantor	Cat# 10062-892

Supplementary Code

MATLAB code for modelling of C2C12 cell differentiation. All rate constants are graphically explained in **Figure S1A,C**.

Model1 in IQM toolbox format:

```
********** MODEL NAME
RASdiff

********** MODEL NOTES
RASdiff, T. Sauter, University of Luxembourg, 01-09/24

********** MODEL STATES
d/dt(MuSC) = r_mP - r_dMuSC
d/dt(TRANS) = r_mPT + r_mT - r_mTD - r_dTRANS
d/dt(DIFF) = 2 * r_mTD - r_dDIFF

MuSC(0) = 981
TRANS(0) = 10249
DIFF(0) = 0

********** MODEL PARAMETERS
sP_PT = 1.16
mT = 1.36
xPT = 1
mTD = 1
d = 1
serumSwitch = 0

********** MODEL VARIABLES
serum = 1 - serumSwitch
PAX7neg = TRANS + DIFF
sumPTD = MuSC + TRANS + DIFF
pMuSC = MuSC/sumPTD * 100
pTRANS = TRANS/sumPTD * 100
pDIFF = DIFF/sumPTD * 100

********** MODEL REACTIONS
```

```
r_mP = sP_PT*(1-xPT) * MuSC *(1+serum)
r_mPT = sP_PT*xPT * MuSC *(1+serum)
r_mT = mT * TRANS *(1+serum)
r_mTD = serumSwitch * mTD * TRANS
r_dMuSC = d * MuSC
r_dTRANS = d * TRANS
r_dDIFF = d * DIFF

********** MODEL FUNCTIONS

********** MODEL EVENTS

********** MODEL MATLAB FUNCTIONS

===
```

Model2 in IQM toolbox format:

```
********** MODEL NAME
RASdiff

********** MODEL NOTES
RASdiff, T. Sauter, University of Luxembourg, 01-10/24

********** MODEL STATES
d/dt(MuSC) = r_mP - r_mPTT - r_dMuSC + r_mPcP - rasDiff*r_mPcP*(fRasCil-1) + rasDiff*(1-min(1,fRasCil))*mPcT
d/dt(TRANS) = r_mT + 2*r_mPTT - r_mTDD - r_dTRANS + r_mPcT + r_mTcT - rasDiff*r_mTcT*(fRasCil-1) + rasDiff*(1-min(1,fRasCil))*mTcD
d/dt(DIFF) = 2*r_mTDD - r_dDIFF + r_mTcD
d/dt(MuSCc) = - r_dMuSCc + rasDiff*r_mPcP*(fRasCil-1) - rasDiff*(1-min(1,fRasCil))*mPcT
d/dt(TRANSc) = - r_dTRANSc + rasDiff*r_mTcT*(fRasCil-1) - rasDiff*(1-min(1,fRasCil))*mTcD

MuSC(0) = 100
TRANS(0) = 7600
DIFF(0) = 0
MuSCc(0) = 400
TRANSc(0) = 1900
```

********** MODEL PARAMETERS

mP = 0

mPTT = 0

mPcP = 0

mPcT = 0

mT = 0

mTDD = 0

mTcT = 0

mTcD = 0

d = 0

serumSwitch = 0

rasDiff=0

fRasCil=1

********** MODEL VARIABLES

serum = 1 - serumSwitch

sumPTD = MuSC + TRANS + DIFF + MuSCc + TRANSc

PAX7pos = MuSC + MuSCc

PAX7neg = TRANS + DIFF + TRANSc

pMuSC = (MuSC+MuSCc)/sumPTD * 100

pTRANS = (TRANS+TRANSc)/sumPTD * 100

pDIFF = DIFF/sumPTD * 100

pCil = (MuSCc+TRANSc)/sumPTD * 100

********** MODEL REACTIONS

r_mP = mP * MuSC *(1+serum)

r_mPTT = mPTT * MuSC *serumSwitch

r_mPcP = mPcP * MuSCc *serum

r_mPcT = mPcT * MuSCc *serumSwitch

r_mT = mT * TRANS *(1+serum)

r_mTDD = mTDD * TRANS *serumSwitch

r_mTcT = mTcT * TRANSc *serum

r_mTcD = mTcD * TRANSc *serumSwitch

r_dMuSC = d * MuSC

r_dMuSCc = d * MuSCc
r_dTRANS = d * TRANS
r_dTRANSc = d * TRANSc
r_dDIFF = d * DIFF

********** MODEL FUNCTIONS

********** MODEL EVENTS

********** MODEL MATLAB FUNCTIONS

Supplementary References

1 Chippalkatti, R. *et al.* RAS isoform specific activities are disrupted by disease associated mutations during cell differentiation. *Eur J Cell Biol* **103**, 151425, doi:10.1016/j.ejcb.2024.151425 (2024).

2 Wall, V. E., Garvey, L. A., Mehalko, J. L., Procter, L. V. & Esposito, D. Combinatorial assembly of clone libraries using site-specific recombination. *Methods Mol Biol* **1116**, 193-208, doi:10.1007/978-1-62703-764-8_14 (2014).

3 Abankwa, D., Gorfe, A. A., Inder, K. & Hancock, J. F. Ras membrane orientation and nanodomain localization generate isoform diversity. *Proc Natl Acad Sci U S A* **107**, 1130-1135, doi:10.1073/pnas.0903907107 (2010).

4 Lavoie, H. *et al.* Inhibitors that stabilize a closed RAF kinase domain conformation induce dimerization. *Nat Chem Biol* **9**, 428-436, doi:10.1038/nchembio.1257 (2013).

5 Schindelin, J. *et al.* Fiji: an open-source platform for biological-image analysis. *Nat Methods* **9**, 676-682, doi:10.1038/nmeth.2019 (2012).

6 Parisi, B., Sunnen, M., Chippalkatti, R. & Abankwa, D. K. A flow-cytometry-based pipeline for the rapid quantification of C2C12 cell differentiation. *STAR Protoc* **4**, 102637, doi:10.1016/j.xpro.2023.102637 (2023).

7 Hao, Y. *et al.* Dictionary learning for integrative, multimodal and scalable single-cell analysis. *Nat Biotechnol* **42**, 293-304, doi:10.1038/s41587-023-01767-y (2024).

8 Trapnell, C. *et al.* The dynamics and regulators of cell fate decisions are revealed by pseudotemporal ordering of single cells. *Nat Biotechnol* **32**, 381-386, doi:10.1038/nbt.2859 (2014).

9 Finak, G. *et al.* MAST: a flexible statistical framework for assessing transcriptional changes and characterizing heterogeneity in single-cell RNA sequencing data. *Genome Biol* **16**, 278, doi:10.1186/s13059-015-0844-5 (2015).

10 Zappia, L. & Oshlack, A. Clustering trees: a visualization for evaluating clusterings at multiple resolutions. *GigaScience* **7**, doi:10.1093/gigascience/giy083 (2018).

11 Blondel, V. D., Guillaume, J.-L., Lambiotte, R. & Lefebvre, E. Fast unfolding of communities in large networks. *Journal of Statistical Mechanics: Theory and Experiment* **2008**, P10008, doi:10.1088/1742-5468/2008/10/P10008 (2008).
