# Supplementary material for: K-Ras controls asymmetric cell divisions from the primary cilium
Source: Cell Death Dis. 2026 Jul 11;17(1):819. doi: 10.1038/s41419-026-09089-y (PMC13594257; doi:10.1038/s41419-026-09089-y)
Supplement: Supplementary file 2 [file 41419_2026_9089_MOESM2_ESM.pdf]

Original Data

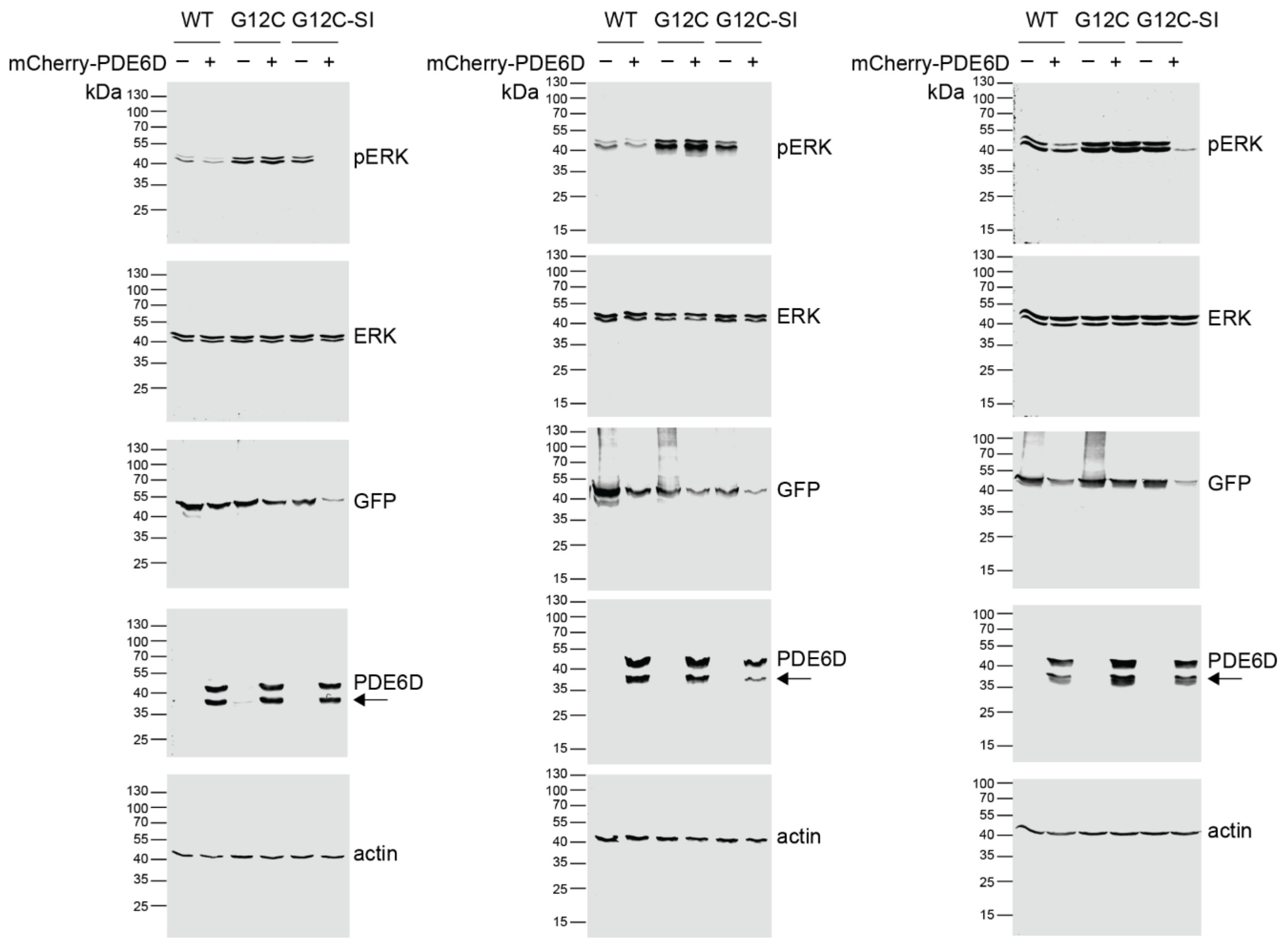

Uncropped Western blot data. Related to Figure S5B.

Whole blots of HEK293 cells transfected with EGFP-K-Ras (WT), EGFP-K-RasG12C (G12C) or EGFP-K-RasG12C-SI (G12C-SI) with or without co-transfection of mCherry-PDE6D at 1:1 DNA ratio for 24 h. Employed antibody labelings are indicated to the right of the blots. Arrows mark truncated/ cleaved protein consistently observed following transfection of mCherry-PDE6D.
